# Integrative computational approach to elucidate the efficacy of cordyceps in managing lupus nephritis: from molecular targets to clinical applications

**DOI:** 10.3389/fmed.2026.1772730

**Published:** 2026-09-07

**Authors:** Chuanchuan Sun, Yanli Du, Ping Pang, Zeping Weng, Yeye Yu, Mingjun Ye, Xinhai Zhao, Heng Shi, Shengyun Sun, Shiping Zhu

**Affiliations:** 1 Department of Traditional Chinese Medicine, The First Affiliated Hospital of Jinan University, Guangzhou, Guangdong Province, China; 2 Department of Nephrology, The First Affiliated Hospital of Jinan University, Guangzhou, Guangdong Province, China; 3 Department of Nephrology, Peking University International Hospital, Beijing, China; 4 Department of Pathology, The First Affiliated Hospital of Jinan University, Guangzhou, Guangdong Province, China; 5 Department of Gastroenterology, The Central Hospital of Shaoyang, Shaoyang, Hunan Province, China; 6 State Key Laboratory of Bioactive Molecules and Druggability Assessment, Department of Traditional Chinese Medicine, The First Affiliated Hospital, Jinan University, Guangzhou, Guangdong Province, China

**Keywords:** biomarker, CFB, cordyceps, lupus nephritis, MAPK1, network pharmacology, therapeutic target

## Abstract

**Background:**

Despite advances in immunosuppressive therapies, lupus nephritis (LN) continues to be a major contributor to morbidity and mortality. The scarcity of reliable biomarkers for predicting renal outcomes further complicates clinical management. While Cordyceps is recognized for its immunomodulatory properties as a medicinal fungus, its therapeutic potential in LN remains underexplored. Moreover, it is unclear whether its molecular targets identified through screening constitute viable therapeutic interventions for this disease.

**Methods:**

Network pharmacology was integrated with LN-associated databases and transcriptomic analysis of GSE200306. Protein–protein interaction, Gene Ontology, and pathway-enrichment analyses were performed to identify candidate targets and biological processes. Molecular docking was used to evaluate predicted interactions between Cordyceps constituents and selected proteins. MAPK1 and CFB expression was further assessed using Nephroseq, an independent transcriptomic cohort (GSE112943), and immunohistochemistry in renal tissues from 30 patients with LN and 10 controls. A multivariable model incorporating age, sex, estimated glomerular filtration rate, MAPK1, and CFB was evaluated using receiver-operating-characteristic, calibration, and decision-curve analyses.

**Results:**

Seven active constituents and 111 putative Cordyceps targets were identified. Intersection with 707 LN-associated genes yielded 26 candidate therapeutic targets enriched in apoptosis, inflammatory responses, complement activation, and NF-*κ*B and PI3K–Akt signaling. Analysis of GSE200306 identified 89 differentially expressed genes, among which MAPK1 and CFB intersected with the Cordyceps–LN target network. Both genes were upregulated in LN kidneys, enriched in the tubulointerstitial compartment, inversely associated with renal function, and supported by immunohistochemistry. The combined model achieved an area under the curve of 0.954 and showed potential clinical utility. In GSE112943, MAPK1 was significantly upregulated, whereas CFB showed a concordant but nonsignificant increase. Docking suggested plausible interactions between peroxyergosterol and MAPK1/CFB but did not establish direct target engagement.

**Conclusions:**

Collectively, these findings identify MAPK1 as a reproducible candidate biomarker and CFB as a compartment-sensitive candidate biomarker in LN. Both molecules represent hypothesis-generating intervention nodes through which Cordyceps-derived constituents may act; direct target-engagement and functional studies are required before they can be considered actionable therapeutic targets.

## Introduction

Chronic kidney disease (CKD) affects more than 10% of the global population and is an increasing cause of morbidity, mortality, and health-care expenditure (1). Irrespective of the initiating disease, persistent epithelial and endothelial injury, maladaptive repair, innate and adaptive immune activation, metabolic dysfunction, fibroblast activation, and excessive extracellular-matrix deposition converge on renal fibrosis and progressive loss of kidney function (2–4). Glomerular and tubular compartments are functionally coupled: disruption of the glomerular filtration barrier exposes tubular cells to filtered proteins, complement components, and cytokines, thereby amplifying oxidative stress, inflammation, and tubulointerstitial fibrosis (3). Lupus nephritis (LN), one of the most serious manifestations of systemic lupus erythematosus (SLE), develops in approximately 40%–60% of patients with SLE, and 10%–15% of patients with LN progress to kidney failure (5, 6). Immune-complex deposition, complement activation, inflammatory-cell recruitment, resident kidney-cell responses, and non-immune CKD drivers jointly determine irreversible nephron loss and long-term outcome in LN (4, 6–8). Although glucocorticoids and immunosuppressive agents remain central to therapy, incomplete kidney responses, cumulative toxicity, infection, relapse, and progression to CKD underscore the need for rigorously validated biomarkers and mechanism-informed therapeutic strategies.

Cordyceps (an Ascomycetes fungus) demonstrates diverse pharmacological properties, including antioxidative, immunomodulatory, and nephroprotective effects (9–11). Its bioactive compounds—such as cordycepin, polysaccharides, sterols, and adenosine—may underlie these activities. Studies indicate Cordyceps ameliorates LN by reducing proteinuria, inflammation, and disease activity (12–14). However, the complexity of traditional formulations obscures definitive identification of bioactive components and targets. To address this, we employed database screening of functional ingredients alongside human validation. This approach clarifies Cordyceps' active constituents and mechanisms in LN treatment, providing a theoretical foundation for clinical application and revealing novel pathogenic insights and therapeutic targets.

## Materials and methods

### Refinement of core drug components and identification of disease targets

To identify Cordyceps' bioactive components and targets, we queried the TCMSP database (15) (https://old.tcmsp-e.com/tcmsp.php) using “Dongchongxiacao” as the keyword. Compounds were filtered by pharmacokinetic criteria (oral bioavailability ≥ 30%; drug-likeness ≥ 0.18). As TCMSP lacks target protein annotations, we retrieved compound structures from PubChem P (16) (https://pubchem.ncbi.nlm.nih.gov/) and submitted their 2D SDF files to PharmMapper (17–19) (https://lilab-ecust.cn/pharmmapper/index.html) for target prediction (Norm Fit > 0.9). Targets obtained from both sources were merged and converted to standardized human gene symbols using UniProt (20) (https://www.uniprot.org/) yielding the final Cordyceps component-target gene set.

We identified LN-associated target genes by searching four databases: GeneCards (21) (https://www.genecards.org/), OMIM (22) (https://www.omim.org/), DisGeNET (23) (https://www.disgenet.org/), and TTD (24) (https://db.idrblab.net/ttd/) using “lupus glomerulonephritis” and “lupus nephritis” as query terms. Genes were filtered by database-specific thresholds: relevance score ≥ 5 (GeneCards) and Score_gda > 0.25 (DisGeNET). After removing duplicates, we merged the results to obtain the final LN target gene set.

We visualized Cordyceps' active ingredients and target genes using Cytoscape 3.9.1. Potential therapeutic targets for LN were identified by intersecting these Cordyceps targets with LN-associated genes. These candidate targets were submitted to the STRING database (https://cn.string-db.org/) to construct a protein-protein interaction (PPI) network. The PPI network was then analyzed in Cytoscape 3.9.1 using the CytoHubba plugin with the MCC algorithm to identify hub genes.

### Gene ontology and pathway enrichment analysis

We performed Gene Ontology (GO) and Kyoto Encyclopedia of Genes and Genomes (KEGG) pathway analyses on the intersecting genes with DAVID database (25). Results were visualized via an online bioinformatics platform (http://www.bioinformatics.com.cn/).

### Analysis of the GSE200306 dataset

To investigate the genetic basis of LN, we downloaded the GSE200306 dataset from the Gene Expression Omnibus (GEO) database (https://www.ncbi.nlm.nih.gov/geo/) and analyzed it using GEO2R to identify differentially expressed genes (DEGs) between LN patients and healthy controls. These DEGs were submitted to the STRING database to generate a PPI network, which was visualized using Cytoscape 3.9.1. We subsequently performed GO enrichment and KEGG pathway analyses through the DAVID database (25), with results visualized via a web-based platform (http://www.bioinformatics.com.cn/).

### Examination of the pathogenesis of LN

Following DAVID analysis of Cordyceps-LN intersecting genes, we identified significant GO terms and KEGG pathways (*P* < 0.05). We applied the same analytical approach to the GSE200306 dataset, yielding comparable GO/KEGG results at identical significance. This convergence suggests shared molecular mechanisms underlying LN pathogenesis and reveals Cordyceps' therapeutic mechanisms against this disease.

### Identification of therapeutic targets of cordyceps for the treatment of lupus nephritis

We performed intersectional analysis between Cordyceps target genes and LN-related genes from multiple sources—including GeneCards, OMIM, DisGeNET, TTD, and the GSE200306 dataset—to delineate specific therapeutic targets modulated by Cordyceps in LN. Concurrently, we investigated Cordyceps' active constituents interacting with these targets.

We validated binding affinities between Cordyceps' primary active constituents and target proteins through molecular docking. Compound structures were downloaded from PubChem in SDF format and converted to PDB format using Open Babel GUI 3.1.1 for ligand preparation. Target protein 3D structures were retrieved from the PDB database (26) (https://www.rcsb.org/) as receptor files. With AutoDockTools 1.5.7, we preprocessed structures by: (1) Removing water molecules; (2) Adding hydrogen atoms; (3) Calculating protein charges; (4) Converting to PDBQT format. Post-docking binding affinity was assessed and visualized in PyMol 3.0.0.

### Discovery of potential predictive targets

To assess clinical relevance, we analyzed candidate genes in the Nephroseq v5 database (https://www.nephroseq.org/) using GraphPad Prism 9.5 for visualization. To generate clinically applicable predictions, we integrated additional clinical factors and candidate genes to develop a multivariate predictive model via R v4.3.1 (rms package).

Control of demographic confounding: Because the LN and control groups differed in age and sex distribution, these variables were prespecified as potential confounders. In the predictive-modeling step, age and sex were entered simultaneously with estimated glomerular filtration rate (eGFR), MAPK1, and CFB in the multivariable model; the coefficients and predicted probabilities for MAPK1 and CFB were therefore conditional on the included demographic and clinical variables. We did not perform propensity-score matching or demographic reweighting. For GSE200306 and GSE112943, complete and harmonized individual-level age, sex, treatment, and histologic-class information was not available for every sample; consequently, the GEO2R differential-expression analyses were not adjusted for these covariates and should be interpreted as group-level associations rather than independent causal effects. We addressed this limitation by reporting the multivariable model separately from the transcriptomic comparisons, performing independent-cohort validation, and explicitly acknowledging residual confounding.

### LN patients and human sample collection and processing

We recruited thirty newly diagnosed LN patients from The First Affiliated Hospital of Jinan University. Control samples comprised histologically normal renal tissues (*n* = 10) obtained from nephrectomy specimens (≥5 cm from lesion margins), with unaffected status confirmed by histopathology. This study was approved by the hospital's Ethics Committee (KY-2025-014) and written informed consent was obtained from all participants. LN patients fulfilled the 2012 Systemic Lupus International Collaborating Clinics (SLICC) classification criteria for SLE and underwent renal biopsy (27). Paraffin-embedded renal tissue sections (4 *μ*m) were deparaffinized, rehydrated, and subjected to antigen retrieval. Non-specific binding was blocked with 5% bovine serum albumin (BSA; Solarbio) for 1 h at room temperature. Sections were probed overnight at 4 °C with primary antibodies specific for MAPK1 (ab32081, 1:50; Abcam) and CFB (ab192577, 1:50; Abcam). After washing, antibody binding was detected using a biotinylated secondary antibody and streptavidin–HRP complex from a commercial SABC-HRP kit (P0615; Beyotime), followed by DAB chromogen (Beyotime) as the substrate. Nuclei were counterstained with hematoxylin. Following dehydration, slides were sealed with neutral mounting medium. Whole-slide digital images were acquired using TissueGnostics scanners, and the percentage of positive cells was quantified with ImageJ software.

## Results

### Acquisition of intersecting genes between cordyceps and LN

**Figure 1:**
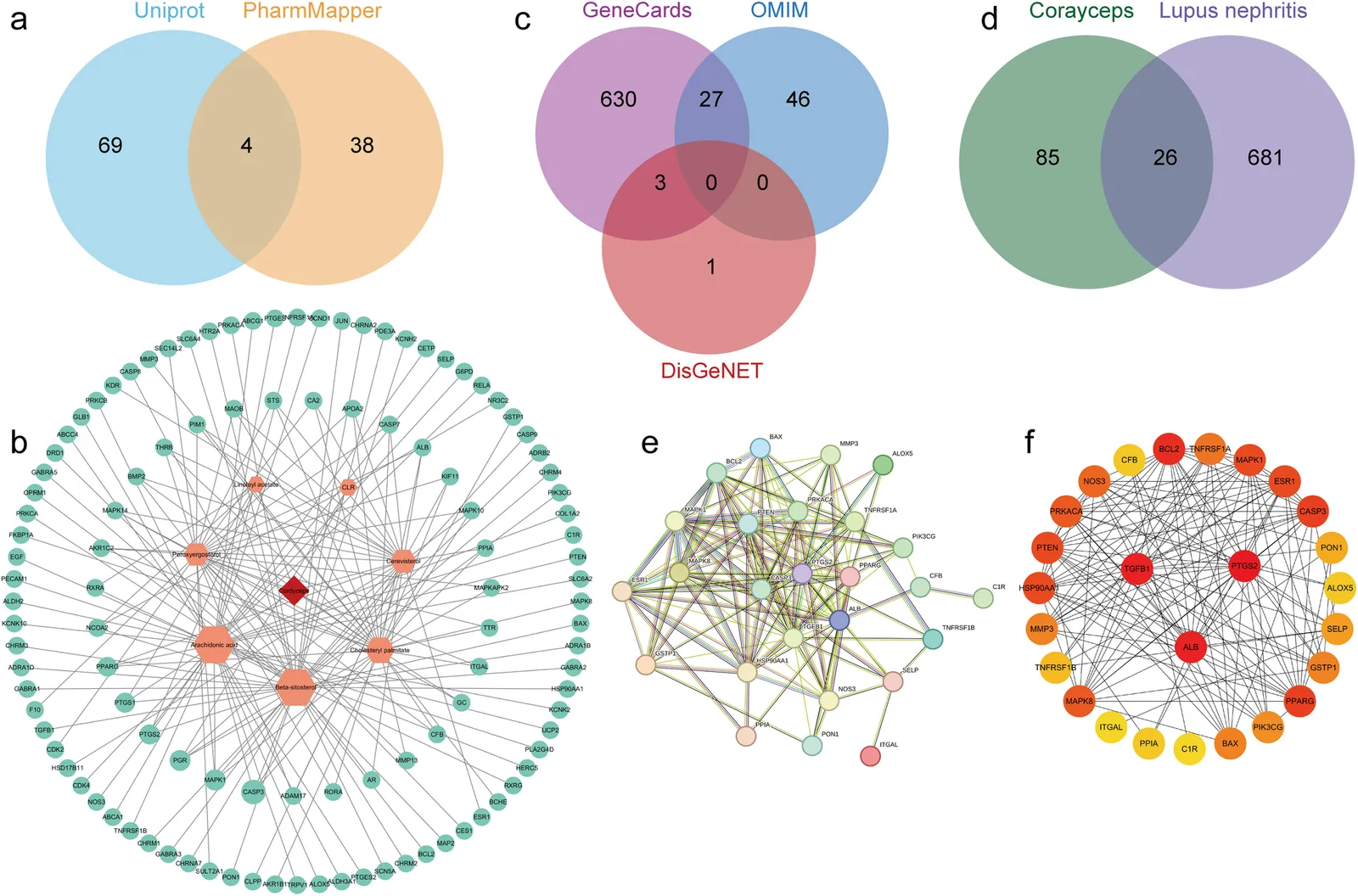
Identification of cordyceps constituents and candidate LN targets. **(a)** Overlap of target genes assigned to Cordyceps constituents by the indicated databases. **(b)** Constituent–target interaction network. **(c)** Overlap of LN-associated genes retrieved from GeneCards, OMIM, and DisGeNET; TTD yielded no eligible targets and is not shown. **(d)** Intersection of Cordyceps targets and LN-associated genes. **(e)** STRING protein–protein interaction network of the 26 intersecting genes (minimum interaction score, 0.400). **(f)** CytoHubba ranking of drug–disease core targets; darker nodes indicate higher network degree. LN, lupus nephritis; PPI, protein–protein interaction; TTD, Therapeutic Target Database.

We had successfully identified seven efficacious constituents of Cordyceps, as delineated in Supplementary Table S1. Subsequent to the standardization process utilizing the UniProt database, a total of 73 molecular targets were procured. Furthermore, employing the PharmMapper server, an additional 42 potential targets were forecasted. Following the elimination of redundant data, a comprehensive list of 111 unique targets was compiled, as depicted in Figure 1a. The interaction between these active constituents and their respective targets was illustrated in Figure 1b.

Database queries identified 600 LN-associated genes from GeneCards, 73 from OMIM, and 4 from DisGeNET. No targets were retrieved from TTD (not shown). After removing duplicates, we obtained 707 unique genes implicated in LN (Figure 1c).

We cross-referenced the Cordyceps targets with LN-associated genes, identifying 26 intersecting genes as potential therapeutic targets for LN (Figure 1d). These genes were used to construct a PPI network using the STRING database (medium confidence: 0.400), resulting in 26 nodes and 140 edges (Figure 1e). Analysis in Cytoscape 3.9.1 ranked the core targets as follows: PTGS2, TGFB1, ALB, BCL2, PPARG, CASP3, MAPK1, ESR1, PTEN, HSP90AA1, PRKACA, MAPK8, NOS3, TNFRSF1A, BAX, GSTP1, MMP3, PIK3CG, SELP, PON1, TNFRSF1B, ALOX5, CFB, PPIA, ITGAL, C1R. The top-ranking genes (PTGS2, TGFB1, ALB) represent the most promising targets for Cordyceps in treating LN.

### Functional annotation

**Figure 2:**
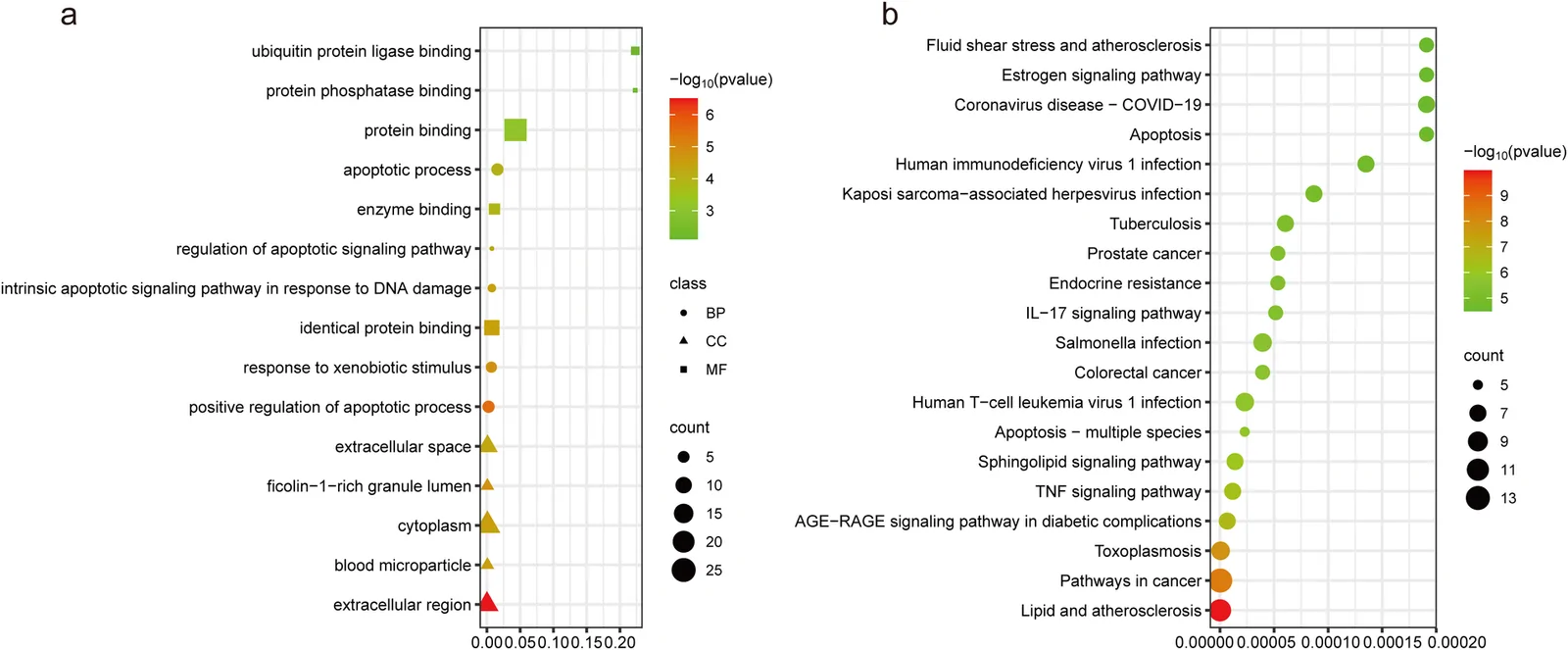
Functional enrichment of the 26 genes shared by cordyceps targets and LN-associated genes. **(a)** Gene Ontology enrichment across biological process, cellular component, and molecular function categories. **(b)** Kyoto Encyclopedia of Genes and Genomes pathway enrichment. The plotted terms are ranked as described in the Methods, and full results are provided in Supplementary Tables S2 and S3. GO, Gene Ontology; KEGG, Kyoto Encyclopedia of Genes and Genomes; LN, lupus nephritis.

GO enrichment analysis revealed the top 5 most significant terms per category (Supplementary Table S2; Figure 2a):Biological Processes (BP): Genes were primarily enriched in positive regulation of apoptosis, response to xenobiotic stimulus, DNA damage-induced intrinsic apoptotic signaling, regulation of apoptotic signaling, and apoptosis. Cellular Components (CC): Key enrichments included extracellular region, ficolin-1-rich granule lumen, cytoplasm, blood microparticle, and extracellular space. Molecular Functions (MF): Significant enrichment occurred in identical protein binding, enzyme binding, protein binding, protein phosphatase binding, and ubiquitin protein ligase binding.

KEGG analysis identified the top 20 enriched pathways (Supplementary Table S3; Figure 2b), including Lipid and atherosclerosis, Pathways in cancer, Toxoplasmosis, AGE-RAGE signaling pathway in diabetic complications, TNF signaling pathway, Sphingolipid signaling pathway, and Apoptosis—multiple species.

### Analysis of the GSE200306 dataset

**Figure 3:**
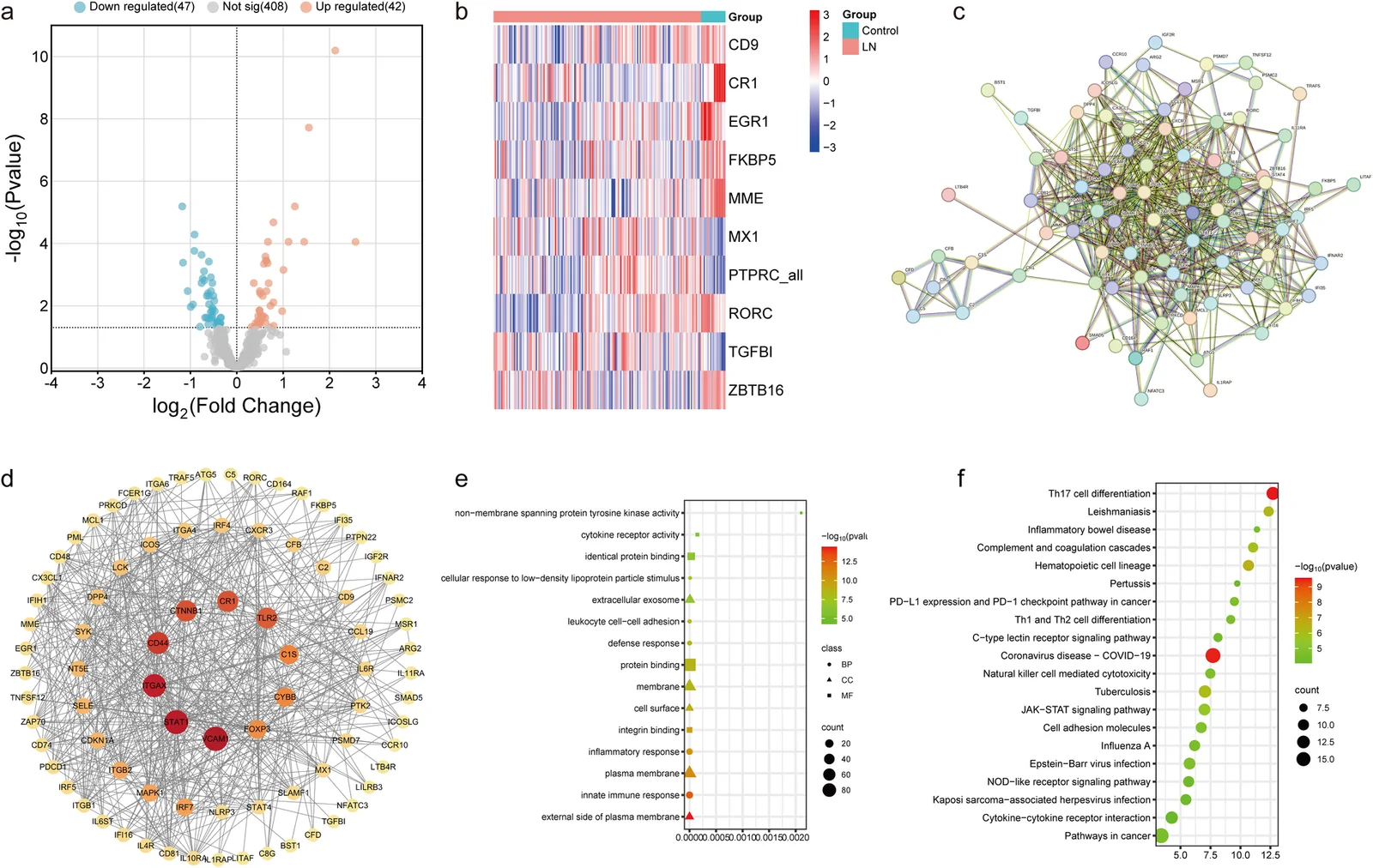
Transcriptomic analysis of GSE200306 (19 controls and 162 LN samples). **(a)** Volcano plot of differential expression. **(b)** Heatmap of genes meeting adjusted *P* < 0.05 and |logFC| ≥ 1. **(c)** STRING PPI network of the 89 differentially expressed genes. **(d)** Cytoscape/CytoNCA ranking by betweenness centrality; larger and darker nodes indicate higher centrality. **(e)** GO enrichment and **(f)** KEGG pathway enrichment of the differentially expressed genes. DEG, differentially expressed gene; GO, Gene Ontology; KEGG, Kyoto Encyclopedia of Genes and Genomes; LN, lupus nephritis; PPI, protein–protein interaction.

Analysis of the GSE200306 dataset (181 samples: 19 controls, 162 LN patients) identified 89 differentially expressed genes (DEGs) between LN patients and controls (Figure 3a), comprising 42 upregulated and 47 downregulated genes. Using stringent criteria (adjusted *P*-value < 0.05, |LogFC| ≥ 1), we generated a heatmap of expression profiles for these filtered DEGs (Figure 3b). Key filtered genes include downregulated PTPRC_all, MX1, and TGFBI, and upregulated CD9, CR1, RORC, FKBP5, MME, EGR1, and ZBTB16.

The 89 differentially expressed genes were analyzed using the STRING database, generating a PPI network (Figure 3c; 81 nodes, 566 edges; PPI enrichment *P* < 1.0 × 10^−16^). This network was imported into Cytoscape 3.9.1 for topological analysis using the CytoNCA plugin. Based on betweenness centrality (BC), the top 10 ranked genes were: VCAM1, STAT1, ITGAX, CD44, CTNNB1, CR1, TLR2, C1S, CYBB, and FOXP3 (Figure 3d). Finally, GO and KEGG enrichment results were visualized using the online platform http://www.bioinformatics.com.cn (Figures 3e,f).

### Investigation into the pathogenesis of LN

Analysis revealed key mechanisms in LN pathogenesis: (1) BP: Involved angiogenesis, apoptosis, inflammatory response, and complement activation. (2) CC: Implicated extracellular region, cytoplasm, plasma membrane, perinuclear region, and extracellular vesicles. (3) MF: Primarily involved protein binding (Supplementary Table S4). KEGG pathway analysis indicated LN development was principally associated with inflammatory pathways, including NF-*κ*B signaling, PI3K-Akt signaling, and Necroptosis (Supplementary Table S5). These molecular findings corroborate the clinical characterization of LN as an autoimmune-mediated inflammatory disorder.

### The tracing of pharmacological targets associated with LN treatment in cordyceps

**Figure 4:**
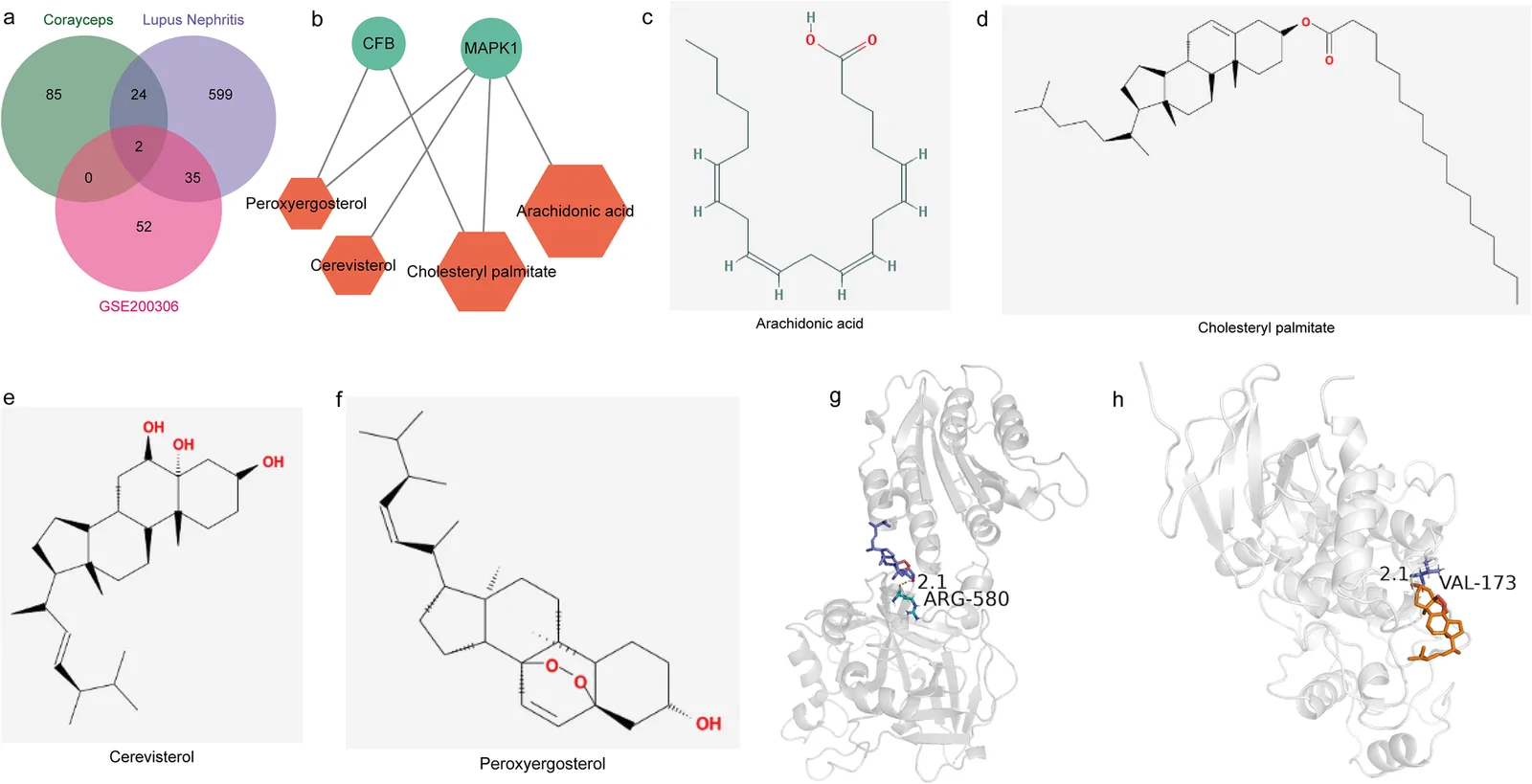
Prioritization and molecular docking of candidate cordyceps targets in LN. **(a)** Intersection of Cordyceps targets, LN-associated genes, and GSE200306 differentially expressed genes. **(b)** Network linking MAPK1 and CFB with predicted active constituents. **(c–f)** Two-dimensional structures of arachidonic acid, cholesteryl palmitate, cerevisterol, and peroxyergosterol obtained from TCMSP. **(g)** Docked pose of peroxyergosterol with CFB (PDB 1RTK). **(h)** Docked pose of peroxyergosterol with MAPK1 (PDB 1TVO). Docking energies are reported in Supplementary Table S6. CFB, complement factor B; LN, lupus nephritis; MAPK1, mitogen-activated protein kinase 1; PDB, Protein Data Bank; TCMSP, Traditional Chinese Medicine Systems Pharmacology Database.

Integrated analysis identified two convergent genes—MAPK1 and CFB (Figure 4a)—through intersection of: (1) Cordyceps sinensis functional targets, (2) LN-associated pathogenic genes, and (3) LN patient vs. healthy control DEGs (GSE200306). Primary active constituents (Arachidonic acid, Cholesteryl palmitate, Cerevisterol, Peroxyergosterol) targeted these genes. Figure 4b shows the pharmacological network, while chemical structures are in Figures 4c–f.

Molecular docking assessed interactions between Peroxyergosterol (a key Cordyceps constituent) and targets CFB/MAPK1. Crystal structures (PDB: 1RTK for CFB; 1TVO for MAPK1) were obtained from the RCSB Protein Data Bank. Docking simulations revealed binding free energies for Peroxyergosterol with each protein (Supplementary Table S6; Figures 4g,h). Molecular docking simulations indicated a plausible interaction interface between peroxyergosterol and MAPK1.

### MAPK1 and CFB may be potential bioindicators for prediction of LN

**Figure 5:**
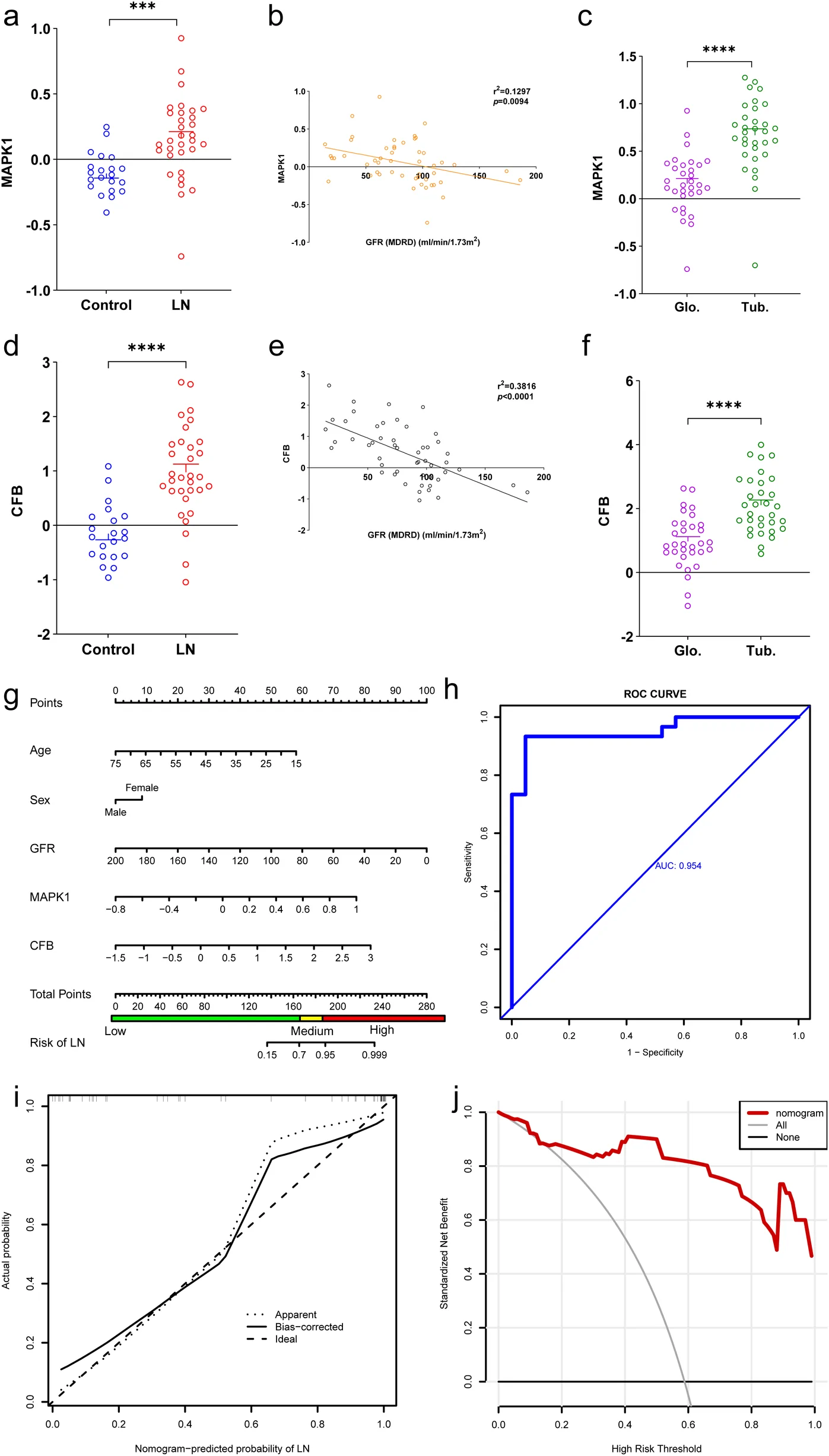
Expression and predictive evaluation of MAPK1 and CFB in LN. **(a)** MAPK1 expression in LN and control kidneys. **(b)** Association between MAPK1 expression and GFR. **(c)** Compartment-specific MAPK1 expression. **(d)** CFB expression in LN and control kidneys. **(e)** Association between CFB expression and GFR. **(f)** Compartment-specific CFB expression. **(g)** Multivariable nomogram incorporating age, sex, eGFR, MAPK1, and CFB. **(h)** Receiver-operating-characteristic curve. **(i)** Calibration plot. **(j)** Decision-curve analysis. Exact sample sizes and statistical procedures are described in the Methods and corresponding source dataset. ***P < 0.001; ****P < 0.0001. CFB, complement factor B; DCA, decision-curve analysis; eGFR, estimated glomerular filtration rate; GFR, glomerular filtration rate; LN, lupus nephritis; MAPK1, mitogen-activated protein kinase 1; ROC, receiver-operating-characteristic.

MAPK1 expression was significantly upregulated in LN patients vs. controls (*P* < 0.001; Figure 5a) and inversely correlated with GFR (Figure 5b). In renal compartments, MAPK1 showed significant elevation specifically in the interstitium (*P* < 0.0001; Figure 5c). Similarly, CFB expression increased in LN kidneys (*P* < 0.0001; Figure 5d), was inversely correlated with GFR (Figure 5e), and showed interstitial enrichment (*P* < 0.0001; Figure 5f). The nomogram incorporated sex, age, eGFR, MAPK1, and CFB, thereby evaluating the molecular markers conditional on these demographic and clinical covariates (Figure 5g). The model achieved an AUC of 0.954 (Figure 5h), with calibration and decision-curve results shown in Figures 5i,j. Immunohistochemistry supported higher MAPK1 and CFB staining in LN renal biopsies (Supplementary Figure S1). Table 1 shows significant differences in sex and age between groups (*P* < 0.001), reflecting the female predominance of LN and the older, predominantly male nephrectomy control population. Accordingly, the model includes age and sex, but residual confounding cannot be excluded, particularly for transcriptomic comparisons that lacked complete harmonized individual-level covariates.

**Table 1:** Baseline characteristics between LN and control.

Variables	Total (*n* = 40)	Con (*n* = 10)	LN (*n* = 30)	*P*
Sex, *n* (%)				<0.001
Female	25 (62)	1 (10)	24 (80)	
Male	15 (38)	9 (90)	6 (20)	
Age (years), Mean ± SD	43.8 ± 16.73	60.2 ± 12.66	38.33 ± 14.25	<0.001
SBP (mmHg), Mean ± SD	125.85 ± 15.23	131 ± 9.9	124.13 ± 16.41	0.125
DBP (mmHg), Mean ± SD	76.62 ± 11.75	73.9 ± 6.26	77.53 ± 13.04	0.249
Scr (μmol/L), Median (Q1,Q3)	94.6 (70.2, 138.42)	90.8 (71.72, 112.62)	94.6 (69.75, 150.15)	0.59
BUN (mmol/L), Median (Q1,Q3)	6.44 (4.8, 10.03)	5.69 (4.95, 6.35)	7.42 (4.85,12.05)	0.111
eGFR (mL/min/1.73m^2^), Mean ± SD	72.69 ± 34.92	76.24 ± 23.93	71.51 ± 38.17	0.649
24hUP (mg/L), Mean ± SD			3,085.33 ± 2,028.94	
C3 (g/L), Mean ± SD			538.12 ± 371.83	
C4 (g/L), Median (Q1,Q3)			104 (52, 179)	
SELENA-SLEDAI Score, Mean ± SD			14.5 ± 5.84	

## Discussion

LN is a major determinant of morbidity, mortality, and CKD progression in SLE (5–7). Diagnosis and classification rely on clinical findings and kidney biopsy because histologic activity and chronicity influence prognosis and treatment. Current regimens combine glucocorticoids with immunosuppressive or targeted agents, yet incomplete response, relapse, cumulative toxicity, and infection remain clinically important (5, 6, 28). The phase 3 REGENCY trial showed that adding obinutuzumab to standard therapy improved complete renal response at week 76, illustrating the value of mechanism-based intervention while also underscoring the need to monitor infectious adverse events (29). These advances strengthen the rationale for discovering new targets, but they do not establish that computationally prioritized molecules are therapeutically actionable without direct target-engagement and functional validation.

Cordyceps, a venerable Chinese medicinal herb, is renowned for its capacity to mitigate oxidative stress-induced damage (14, 30–32), curtail inflammatory responses (13, 31–34), and ameliorate kidney fibrosis (13, 14, 31, 35–37). Cordyceps had been shown to effectively mitigate lupus activity and proteinuria in MRL/lpr mice, a model for systemic lupus erythematosus (SLE). These therapeutic effects were attributed to the extract's capacity to negatively modulate the STAT3/mTOR/NF-*κ*B signaling cascades. Consequently, there was a significant reduction in the levels of pathogenic anti-dsDNA antibodies, antinuclear antibodies (ANA), and blood urea nitrogen (BUN), concomitant with an increase in the complement component C3. These changes culminated in a marked decrease in urinary protein excretion and the urine albumin-to-creatinine ratio (ACR). The treatment also resulted in a notable inhibition of renal macrophage infiltration and glomerular mesangial cell proliferation, hallmark features of LN. By downregulating *α*-SMA and TGF-*β*1, the extract substantially ameliorated renal fibrosis in LN mice and curtailed cellular apoptosis (12, 13). In a separate line of investigation, it had been elucidated that Cordyceps exerted its renoprotective effects by targeting the PI3K/mTOR-mediated autophagy pathway in the kidneys of MRL/lpr mice, thereby attenuating inflammatory responses, oxidative stress, and renal fibrosis (14). Our findings demonstrated that MAPK1 and CFB represented promising therapeutic targets for Cordyceps -mediated intervention in LN. Furthermore, their dysregulated expression profiles suggested utility as diagnostic biomarkers for the condition.

In 1994, researchers initially delineated the Mitogen-Activated Protein Kinases (MAPKs), identifying ERK, p38, and JNK as pivotal pathways within the MAPK network, particularly activated during instances of inflammation and stress (38). Mitogen-activated protein kinase 1 (MAPK1) plays a critical role in the MAPK/ERK cascade, orchestrating diverse cellular processes, including proliferation, differentiation, transcription, apoptosis, and stress responses (39). The activation of MAPK1 had been linked to the preservation of renal function during ischemia-reperfusion kidney injury (40). Investigations had indicated that the acute tubular epithelial cell death induced by cisplatin was mediated through the MAPK pathway (41). Furthermore, MAPK1 had been identified as a key factor in the development and progression of diabetic nephropathy. Hyperglycemic conditions can trigger the MAPK1 signaling pathway, leading to glomerular hyperplasia, thickening of the basement membrane, and mesangial cell proliferation all of which exacerbate diabetic nephropathy (42). Concurrently, the destruction of the mitochondria-associated endoplasmic reticulum membrane (MAM) in diabetic nephropathy was significantly associated with the upregulation of MAPK1 and the downregulation of PACS-2. Functional studies had revealed that inhibiting the upregulation of MAPK1 and enhancing PACS-2 expression can substantially prevent the degradation of MAM integrity, reduce proteinuria, and ameliorate pathological renal damage in diabetic mice (43). Additionally, the increased resistance to cyclosporine A may stem from the activation of the ERK1/2 MAPK pathway, which resulted in decreased permeability of renal epithelial cells (44). In patients with LN, the P38 MAPK pathway contributed to disease progression by promoting pro-inflammatory cytokines IL-10 and MCP-1. The administration of SB203580, a specific p38 MAPK inhibitor, had been shown to suppress the secretion of these inflammatory factors (45). Consistent with our research findings, the activation of MAPK1 may propel the advancement of LN, and cordyceps may offer renal protection by inhibiting MAPK1.

Complement factor B (CFB), a pivotal serine protease of the alternative complement pathway, is predominantly synthesized and secreted by hepatocytes. In healthy kidneys, its expression is typically undetectable or confined to areas of renal tubules affected by interstitial inflammation (46). Investigations have revealed that congenital nephrotic syndrome, diabetic nephropathy, and lupus nephritis demonstrated positive staining for CFB dominantly in the peritubular regions, whereas membranous nephropathy exhibited a negative result (46, 47). Research has found that the onset of LN is closely related to the abnormal activation of the complement alternative pathway (46–50), and the enablement of the complement pathway mediated by CFB can exacerbate LN damage (51, 52). The initiation of CFB may directly damage the renal tubules, recruit inflammatory mediators to the peri-tubular environment, induce renal tubulointerstitial injury, and these peri-tubular inflammations and edema may slowly progress to fibrosis and tubular atrophy. Renal tubulointerstitial injury served as a predictor of advancement from glomerulonephritis to end-stage renal disease (46). A deficiency in factor B or the administration of LNP023 (an inhibitor of complement factor B) can ameliorate the manifestations of lupus nephritis in MRL/lpr mice by modulating the expression of PI3K-Akt, HIF-1, and ErbB (53).

This intervention reduced serum levels of ANA and anti-dsDNA antibodies, diminished renal deposition of complement and antibody immune complexes (ICs), and attenuated crescent formation, fibrinoid necrosis, and inflammatory cell infiltration, ultimately leading to decreased proteinuria and conferring a protective benefit on the kidneys (51, 53). In accordance with literature (47), our study discovered that CFB expression was minimal in the kidneys of healthy individuals but significantly elevated in LN patients. Upon comparing CFB expression in the glomeruli and renal tubules of LN patients, we observed a particularly notable upregulation in the renal tubules. This suggested that the kidney's synthesis of CFB primarily occurred in the renal tubules (46). Furthermore, our findings suggested that Cordyceps sinensis may mediate therapeutic benefits in LN via modulating CFB activity.

Our analytical framework integrates network pharmacology with transcriptomic profiling to validate therapeutic targets, aligning with contemporary strategies for target discovery and verification. MAPK1 and CFB constitute core nodes in inflammatory signaling: MAPK1 governs intracellular signal transduction, whereas CFB mediates the complement cascade. By targeting these two molecules, Cordyceps may reshape the renal inflammatory microenvironment and thereby exert therapeutic effects on LN. A recent study by Zhong et al. employed a conceptually analogous approach—integrating transcriptomics, gene knockout, and therapeutic targeting—to identify MrgD as a novel modeling and therapeutic target in pulmonary arterial hypertension (54). This paradigm of unbiased transcriptomic screening coupled with experimental validation provides a strong conceptual foundation for our target identification pipeline and reinforces the notion that MAPK1 and CFB represent credible intervention nodes in LN. Central to the proposed renoprotective effects of Cordyceps is the concept of microenvironmental preservation. Yao et al. recently developed an injectable biomimetic microtissue system that enhances stem cell survival and differentiation in the hostile post-infarction myocardial microenvironment by modulating local inflammatory and fibrotic signaling (55). The conceptual parallel is compelling: just as this engineered niche rescues therapeutic cells by recalibrating the local signaling milieu, Cordyceps-derived constituents may exert renal protection by rebalancing inflammatory and fibrotic signals within the tubulointerstitial microenvironment. Notably, we found that MAPK1 and CFB are predominantly localized to renal tubules in LN kidneys, further suggesting that Cordyceps may alleviate LN by modulating key molecules within the tubulointerstitial microenvironment. These references anchor our mechanistic reasoning in the broader landscape of translational biomedical research and underscore the therapeutic rationale of microenvironment-targeted interventions in LN.

Integrating our KEGG enrichment and docking results with recent kidney-disease studies suggests a testable, but unproven, framework in which Cordyceps-derived constituents converge on MAPK1- and CFB-centred inflammatory circuits. MAPK1/ERK could couple TNF and other stress inputs to NF-*κ*B-dependent cytokine production, mitochondrial reactive oxygen species, and TGF-*β*/Smad- and Wnt1/*β*-catenin-driven fibroblast activation. Consistent with this model, studies in glomerular disease implicate AhR-dependent oxidative inflammation, TXNIP-NLRP3 activation, a YY1-HIF-1*α*-mROS feed-forward loop, Wnt1/*β*-catenin-mediated podocyte injury and HIF-1*α*/HO-1-associated ferroptosis as mutually reinforcing drivers of renal injury (56–61). In parallel, CFB may increase alternative-pathway C3 convertase activity and local complement amplification, linking immune-complex injury to tubulointerstitial inflammation and glomerular-tubular crosstalk (3, 8), while microbiota-dependent tryptophan metabolism may tune AhR-Wnt/*β*-catenin signalling and thereby couple metabolic dysregulation to fibrosis (62). Although docking predicts interactions of peroxyergosterol with MAPK1 and CFB, it cannot establish ATP-pocket occupancy, kinase inhibition, or complement modulation. These associations therefore define a testable mechanistic hypothesis rather than an established mode of action. Future studies should combine direct binding and kinase assays with pathway and functional readouts, MAPK1 and CFB gain- and loss-of-function approaches, renal-cell co-culture systems and *in vivo* LN models to establish target engagement and causality for defined Cordyceps-derived constituents.

Furthermore, we re-analyzed the independent LN transcriptomic dataset GSE112943, which contains renal-tissue samples from 14 patients with LN and 7 healthy controls. MAPK1 showed concordant and statistically significant upregulation (logFC = 3.25, *P* < 0.0001), supporting cross-cohort reproducibility. CFB showed a directionally concordant but smaller effect (logFC = 1.48, *P* = 0.064) and did not meet the conventional *P* < 0.05 threshold. The absence of statistical significance does not prove absence of biological involvement, but it materially weakens claims of universal overexpression. Plausible sources of heterogeneity include limited power in the small validation cohort, platform and probe-specific dynamic range, preprocessing differences, dilution of a tubulointerstitial signal in whole-kidney tissue, and variation in histologic class, activity/chronicity, treatment exposure, ancestry, age, sex, and cellular composition. Complement activation is biologically relevant to immune-complex kidney injury (8), but the present cross-cohort data support CFB only as a compartment- and context-sensitive candidate biomarker. Larger cohorts with harmonized clinical annotation and cell- or compartment-resolved expression are required before clinical generalization.

In summary, our findings indicate that Cordyceps-derived constituents may exert modulatory effects on MAPK1/CFB-associated signaling axes, implicating these molecular hubs as candidate therapeutic vulnerabilities in LN. However, several limitations should be noted. First, the lack of direct biochemical validation—specifically, *in vitro* kinase assays for MAPK1 phosphorylation under Cordyceps treatment—precludes definitive mechanistic conclusions. Although our integrative analyses identify MAPK1 and CFB as disease-associated biomarkers and candidate intervention nodes, confirmation of Cordyceps constituents as bona fide kinase inhibitors requires future gain- or loss-of-function studies. Second, the predominantly adult composition of our cohort limits generalizability to pediatric-onset SLE, a condition distinguished by heightened genetic burden, more frequent renal involvement, accelerated disease onset, distinct treatment responses, and higher relapse rates (63, 64). Multicenter prospective cohorts and functional experimental validation are warranted to further substantiate our findings.

## Conclusion

Collectively, our findings support MAPK1 as a reproducible candidate biomarker and identify CFB as a promising candidate in LN, although its expression appears sensitive to cohort composition. Network and docking analyses suggest that these molecules may participate in the response to Cordyceps-derived constituents, but the present data do not establish direct target engagement or therapeutic action. Larger demographically balanced cohorts and mechanistic experiments are needed to validate predictive performance and causality.

## Data Availability

The original contributions presented in the study are included in the article/Supplementary Material, further inquiries can be directed to the corresponding author/s.
