# Supplementary material for: Integrative computational approach to elucidate the efficacy of cordyceps in managing lupus nephritis: from molecular targets to clinical applications
Source: Front Med (Lausanne). 2026 Sep 7;13:1772730. doi: 10.3389/fmed.2026.1772730 (PMC13590306; doi:10.3389/fmed.2026.1772730)
Supplement: Supplementary file 3 [file Table2.docx]

**Supplementary Tables**

**Table 1.**

**Table 1.The main components of Cordyceps**

| Mol ID | Molecule Name | MW | OB (%) | DL |
| --- | --- | --- | --- | --- |
| MOL001439 | Arachidonic acid | 304.52 | 45.57 | 0.2 |
| MOL001645 | Linoleyl acetate | 308.56 | 42.1 | 0.2 |
| MOL000358 | beta-Sitosterol | 414.79 | 36.91 | 0.75 |
| MOL011169 | Peroxyergosterol | 428.72 | 44.39 | 0.82 |
| MOL008998 | Cerevisterol | 432.76 | 39.52 | 0.77 |
| MOL008999 | Cholesteryl palmitate | 625.19 | 31.05 | 0.45 |
| MOL000953 | CLR | 386.73 | 37.87 | 0.68 |

**Table 2.**

**Table 2. GO terms and enrichment analysis of genes**

| GO ID | Description | PValue | Fold Enrichment | Count |
| --- | --- | --- | --- | --- |
| BP |  |  |  |  |
| GO:0043065 | positive regulation of apoptotic process | 2.78E-06 | 16.15049125 | 7 |
| GO:0009410 | response to xenobiotic stimulus | 1.52E-05 | 17.77476923 | 6 |
| GO:0008630 | intrinsic apoptotic signaling pathway in response to DNA damage | 3.22E-05 | 61.71794872 | 4 |
| GO:2001233 | regulation of apoptotic signaling pathway | 3.38E-05 | 317.4065934 | 3 |
| GO:0006915 | apoptotic process | 9.02E-05 | 8.727790728 | 7 |
| CC |  |  |  |  |
| GO:0005576 | extracellular region | 3.08E-07 | 5.087370732 | 14 |
| GO:1904813 | ficolin-1-rich granule lumen | 1.50E-05 | 31.57076923 | 5 |
| GO:0005737 | cytoplasm | 2.82E-05 | 2.520732107 | 18 |
| GO:0072562 | blood microparticle | 2.84E-05 | 26.8458922 | 5 |
| GO:0005615 | extracellular space | 4.62E-05 | 4.496096084 | 11 |
| MF |  |  |  |  |
| GO:0042802 | identical protein binding | 3.73E-05 | 4.607244255 | 11 |
| GO:0019899 | enzyme binding | 1.14E-04 | 11.65137803 | 6 |
| GO:0005515 | protein binding | 6.54E-04 | 1.42887627 | 25 |
| GO:0019903 | protein phosphatase binding | 0.006141312 | 24.48098531 | 3 |
| GO:0031625 | ubiquitin protein ligase binding | 0.007849738 | 9.311143984 | 4 |

**Table 3.**

**Table 3. KEGG pathways enrichment analysis of genes**

| KEGG pathway | Description | Fold Enrichment | PValue | Count |
| --- | --- | --- | --- | --- |
| hsa05417 | Lipid and atherosclerosis | 17.72688372 | 1.04E-10 | 11 |
| hsa05200 | Pathways in cancer | 8.482561205 | 3.42E-09 | 13 |
| hsa05145 | Toxoplasmosis | 24.97153153 | 1.36E-08 | 8 |
| hsa04933 | AGE-RAGE signaling pathway in diabetic complications | 24.2536 | 2.32E-07 | 7 |
| hsa04668 | TNF signaling pathway | 21.27508772 | 5.05E-07 | 7 |
| hsa04071 | Sphingolipid signaling pathway | 20.04429752 | 7.19E-07 | 7 |
| hsa04215 | Apoptosis - multiple species | 54.1375 | 1.55E-06 | 5 |
| hsa05166 | Human T-cell leukemia virus 1 infection | 12.48576577 | 1.58E-06 | 8 |
| hsa05210 | Colorectal cancer | 24.17302326 | 3.14E-06 | 6 |
| hsa05132 | Salmonella infection | 11.13188755 | 3.40E-06 | 8 |
| hsa04657 | IL-17 signaling pathway | 22.11574468 | 4.88E-06 | 6 |
| hsa05215 | Prostate cancer | 21.43175258 | 5.70E-06 | 6 |
| hsa01522 | Endocrine resistance | 21.21306122 | 6.00E-06 | 6 |
| hsa05152 | Tuberculosis | 13.47422222 | 7.30E-06 | 7 |
| hsa05167 | Kaposi sarcoma-associated herpesvirus infection | 12.50185567 | 1.12E-05 | 7 |
| hsa05170 | Human immunodeficiency virus 1 infection | 11.44037736 | 1.86E-05 | 7 |
| hsa04210 | Apoptosis | 15.28588235 | 2.96E-05 | 6 |
| hsa04915 | Estrogen signaling pathway | 15.17430657 | 3.07E-05 | 6 |
| hsa05171 | Coronavirus disease - COVID-19 | 10.40927039 | 3.18E-05 | 7 |
| hsa05418 | Fluid shear stress and atherosclerosis | 14.95597122 | 3.29E-05 | 6 |

**Table 4.**

**Table 4. Results from the GO Analysis of the Tripartite Intersections**

| Term | Description |
| --- | --- |
| BP |  |
| GO:0001525 | angiogenesis |
| GO:0006915 | apoptotic process |
| GO:0006954 | inflammatory response |
| GO:0006956 | complement activation |
| GO:0007159 | leukocyte cell-cell adhesion |
| GO:0007165 | signal transduction |
| GO:0008285 | negative regulation of cell proliferation |
| GO:0009410 | response to xenobiotic stimulus |
| GO:0031663 | lipopolysaccharide-mediated signaling pathway |
| GO:0032496 | response to lipopolysaccharide |
| GO:0032930 | positive regulation of superoxide anion generation |
| GO:0043065 | positive regulation of apoptotic process |
| GO:0045087 | innate immune response |
| GO:0050728 | negative regulation of inflammatory response |
| GO:0051897 | positive regulation of protein kinase B signaling |
| GO:0071404 | cellular response to low-density lipoprotein particle stimulus |
| GO:0097191 | extrinsic apoptotic signaling pathway |
| GO:0098586 | cellular response to virus |
| CC |  |
| GO:0005576 | extracellular region |
| GO:0005615 | extracellular space |
| GO:0005737 | cytoplasm |
| GO:0005829 | cytosol |
| GO:0005886 | plasma membrane |
| GO:0009986 | cell surface |
| GO:0032991 | macromolecular complex |
| GO:0048471 | perinuclear region of cytoplasm |
| GO:0070062 | extracellular exosome |
| GO:1904813 | ficolin-1-rich granule lumen |
| MF |  |
| GO:0042802 | identical protein binding |
| GO:0005515 | protein binding |

**Table 5.**

**Table 5. KEGG Results for the Tripartite Intersections**

| Term | Description |
| --- | --- |
| hsa04064 | NF-kappa B signaling pathway |
| hsa04151 | PI3K-Akt signaling pathway |
| hsa04217 | Necroptosis |
| hsa04380 | Osteoclast differentiation |
| hsa04510 | Focal adhesion |
| hsa04621 | NOD-like receptor signaling pathway |
| hsa04625 | C-type lectin receptor signaling pathway |
| hsa04650 | Natural killer cell mediated cytotoxicity |
| hsa04659 | Th17 cell differentiation |
| hsa04664 | Fc epsilon RI signaling pathway |
| hsa04668 | TNF signaling pathway |
| hsa04933 | AGE-RAGE signaling pathway in diabetic complications |
| hsa05020 | Prion disease |
| hsa05131 | Shigellosis |
| hsa05133 | Pertussis |
| hsa05140 | Leishmaniasis |
| hsa05144 | Malaria |
| hsa05145 | Toxoplasmosis |
| hsa05146 | Amoebiasis |
| hsa05150 | Staphylococcus aureus infection |
| hsa05152 | Tuberculosis |
| hsa05160 | Hepatitis C |
| hsa05161 | Hepatitis B |
| hsa05162 | Measles |
| hsa05163 | Human cytomegalovirus infection |
| hsa05164 | Influenza A |
| hsa05165 | Human papillomavirus infection |
| hsa05166 | Human T-cell leukemia virus 1 infection |
| hsa05167 | Kaposi sarcoma-associated herpesvirus infection |
| hsa05169 | Epstein-Barr virus infection |
| hsa05170 | Human immunodeficiency virus 1 infection |
| hsa05171 | Coronavirus disease - COVID-19 |
| hsa05200 | Pathways in cancer |
| hsa05203 | Viral carcinogenesis |
| hsa05205 | Proteoglycans in cancer |
| hsa05210 | Colorectal cancer |
| hsa05212 | Pancreatic cancer |
| hsa05213 | Endometrial cancer |
| hsa05216 | Thyroid cancer |
| hsa05222 | Small cell lung cancer |
| hsa05417 | Lipid and atherosclerosis |

**Table 6.**

**Table 6. Molecular docking results**

| Pubchem ID | Molecular Formula | Ligands | Target | Binding energy（kcal.mol-1） | PBD |
| --- | --- | --- | --- | --- | --- |
| 5351516 | C28H44O3 | Peroxyergosterol | CFB | -4.69 | 1RTK |
| 5351516 | C28H44O3 | Peroxyergosterol | MAPK1 | -5.3 | 1TVO |
