# Supplementary material for: Integrative computational approach to elucidate the efficacy of cordyceps in managing lupus nephritis: from molecular targets to clinical applications
Source: Front Med (Lausanne). 2026 Sep 7;13:1772730. doi: 10.3389/fmed.2026.1772730 (PMC13590306; doi:10.3389/fmed.2026.1772730)
Supplement: Supplementary file 5 [file Table4.docx]

**Supplementary Figure**

Figure 1. Expression levels of MAPK1 and CFB in human renal tissues (Magnification, ×20). a-b. Immunohistochemistry and analysis of MAPK1 in human kidney. c-d. Immunohistochemistry and analysis of CFB in human kidney. Con: Control, LN: Lupus Nephritis.(***p<0.001.****p<0.0001)
