# Supplementary material for: Integrative computational approach to elucidate the efficacy of cordyceps in managing lupus nephritis: from molecular targets to clinical applications
Source: Front Med (Lausanne). 2026 Sep 7;13:1772730. doi: 10.3389/fmed.2026.1772730 (PMC13590306; doi:10.3389/fmed.2026.1772730)
Supplement: Supplementary file 6 [file Table8.docx]

The binding free energies for the molecular docking of peroxyergosterol with MAPK1 and CFB are shown in the table below:

| Pubchem ID | Molecular Formula | Ligands | Target | Binding energy（kcal.mol-1） | PBD |
| --- | --- | --- | --- | --- | --- |
| 5351516 | C28H44O3 | Peroxyergosterol | CFB | -4.69 | 1RTK |
| 5351516 | C28H44O3 | Peroxyergosterol | MAPK1 | -5.3 | 1TVO |
