# Supplementary material for: Integrative computational approach to elucidate the efficacy of cordyceps in managing lupus nephritis: from molecular targets to clinical applications
Source: Front Med (Lausanne). 2026 Sep 7;13:1772730. doi: 10.3389/fmed.2026.1772730 (PMC13590306; doi:10.3389/fmed.2026.1772730)
Supplement: Supplementary file 7 [file Table3.docx]

**Original Image for Supplementary Figure 1**

**a** Con

LN

c Con

LN
